# The Diversity of *Coolia* spp. (Dinophyceae Ostreopsidaceae) in the Central Great Barrier Reef Region

**DOI:** 10.1371/journal.pone.0079278

**Published:** 2013-10-23

**Authors:** Paolo Momigliano, Leanne Sparrow, David Blair, Kirsten Heimann

**Affiliations:** 1 School of Marine and Tropical Biology, James Cook University, Townsville, Queensland, Australia; 2 Centre for Sustainable Tropical Fisheries and Aquaculture, James Cook University, Townsville, Queensland, Australia; 3 North Queensland Algal Identification/Culturing Facility, James Cook University, Townsville, Queensland, Australia; American University in Cairo, Egypt

## Abstract

**Background:**

Dinoflagellates are important primary producers, crucial in marine food webs. Toxic strains, however, are the main causative agents of non-bacterial seafood poisoning, a major concern for public health worldwide. Despite their importance, taxonomic uncertainty within many genera of dinoflagellates is still high. The genus *Coolia* includes potentially harmful species and the diversity within the genus is just starting to become apparent.

**Methodology/Principal Findings:**

In the current study, cultures were established from strains of *Coolia* spp. isolated from the central Great Barrier Reef (GBR). Cultures were identified based on thecal plate morphology and analyses of sequences (18S, ITS and 28S) from the nuclear rRNA operon. We report that the central GBR harbors a high diversity of *Coolia* species, including two species known to be capable of toxin production (*C. tropicalis* and *C. malayensis*), as well as the non-toxic *C. canariensis*. The strain of *C. canariensis* isolated from the GBR may in fact be a cryptic species, closely related but nevertheless phylogenetically distinct from the strain on which the holotype of *C. canariensis* was based. We also found evidence of the occurrence of a cryptic species morphologically very similar to both *C. malayensis* and *C. monotis*. The consequences of taxonomic confusion within the genus are discussed.

**Conclusion/Significance:**

The central GBR region harbors a previously unreported high diversity of *Coolia* spp., including two species known to potentially produce toxins. The presence of a cryptic species of unknown toxicity highlights the importance of cryptic diversity within dinoflagellates.

## Introduction

Marine dinoflagellates have been extensively studied for a number of reasons. They are important primary producers, and may on occasion dominate planktonic and benthic microalgal communities [1,2]. Furthermore, the symbiotic relationships between certain dinoflagellates (*Symbiodinium* spp.) and marine invertebrates allows coral reefs to thrive in oligotrophic tropical waters [3,4]. Dinoflagellates, however, are also a major concern for public health. Toxic strains are the main causative agents of non-bacterial seafood poisonings, and a number of benthic genera (such as *Gambierdiscus*, *Coolia*, *Prorocentrum, Ostreopsis* and *Amphidinium*) include species that are able to produce toxins [5-9].

Despite their impact on public health, the taxonomy of many benthic dinoflagellates remains largely unexplored. A sound understanding of dinoflagellate diversity is essential for monitoring potentially toxic strains, as closely related species, and even strains within the same species, may differ in toxicity [10,11] and climate-induced changes in sea surface temperatures may alter distribution patterns [12]. Armored dinoflagellates are traditionally identified based on thecal plate morphology, revealed by light microscopy and scanning electron microscopy (SEM); however, the possibility of morphologically cryptic species and/or phenotypic plasticity, the latter induced by environmental factors, creates obvious difficulties. A molecular phylogenetic approach is therefore often used to supplement morphology in species description and identification, particularly when morphological differences appear to be small [13-22]. Phylogenetic reconstructions are usually based on genes of the rRNA operon (18S, 28S and 5.8S rRNA) as they are thought to be sufficiently variable to provide information on species-level divergence and the ITS region shows a clear gap between intra- and inter-specific distances making it an ideal marker for distinguishing between closely related taxa and to identify cryptic species [18,23].

Prior to the application of molecular techniques, the genus *Gambierdiscus* was thought to consist of a single species, *G. toxicus*, with a cosmopolitan distribution and considered to be principally responsible for ciguatera fish poisoning (CFP) [5]. Recent studies, utilizing molecular phylogenetics, showed that the monotypic genus *Gambierdiscus* actually consisted of more than 10 species, not all of which may be toxic [13,18,24,25]. The genus *Coolia* also contains toxic species [9,26,27] and co-occurrence with *G. toxicus* has been observed in areas with endemic CFP [28]. Following the description of *Coolia monotis* in 1919 [29], this genus remained monotypic for almost 90 years. Since 1995, four additional species have been described: *C. areolata, C. tropicalis*, *C. canariensis* and *C. malayensis* [14,17,30-32]. At present, molecular data are available for the last three of these species. Acquisition of more molecular data may reveal the presence of cryptic species, a phenomenon that has important repercussions in terms of monitoring harmful species, as closely related taxa which are morphologically very similar may differ in toxicity. 

The diversity of *Coolia* spp. in the central region of the Great Barrier Reef (GBR), an area with endemic CFP, is unknown, as no published records are available. In this study we give a first report on the diversity of *Coolia* spp. in the central GBR. Strains of *Coolia* spp. were isolated, taken into culture and their taxonomic position established using a combination of morphological and phylogenetic analyses. Regions of the rRNA operon (18S and 28S genes and the ITS region) were used for phylogenetic analysis. We discovered that the central GBR harbors a high diversity of *Coolia* species, including three known species (*C. malayensis*, *C. tropicalis* and *C. canariensis*) as well as a cryptic species which is morphologically very similar to *C. monotis* and *C. malayensis* but phylogenetically very distinct. A second cryptic species closer to *C. canariensis* might also exist. The high molecular diversity of *Coolia* spp. in the central GBR is described and the problems generated by taxonomic confusion within this taxon are discussed. 

## Results

Based on morphology and molecular data, four strains of *Coolia* spp. were isolated and cultured from GBR waters. Three of these were identified based on morphology and molecular data as *C. malayensis* (NQAIF35), *C. canariensis* (NQAIF252) and *C. tropicalis* (NQAIF90). One culture (NQAIF103) is morphologically very similar to both *C. malayensis* and *C. monotis*, however it is clearly distinct based on genetic data. A detailed morphological description of the strain NQAIF103 for comparison with the closely related *C. malyensis* and *C. monotis* is provided. A morphological description of the strain NQAIF90 (*C. tropicalis*) is also given, to allow comparison with the original description [30] and recent re-description of this species [32]. 

### DNA sequence analysis

**Figure 1:**
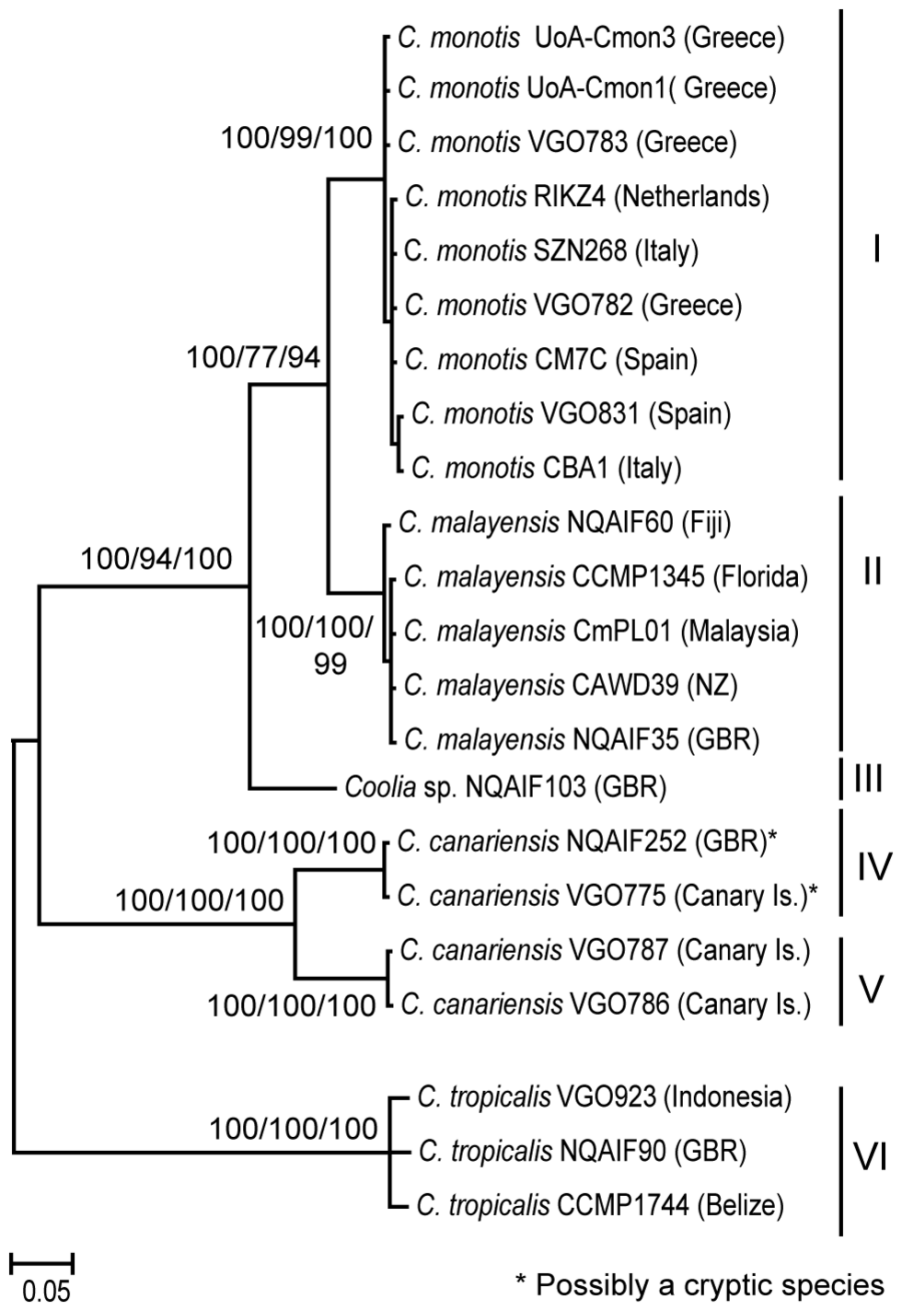
Phylogeny of the genus *Coolia* based on available 28S rRNA gene sequences. Six distinct clades are identified (Roman numerals). Branch labels indicate Bayesian clade credibility values, and MP and ML bootstrap support, respectively. The tree was midpoint rooted. Scale bar represent number of changes.

**Figure 2:**
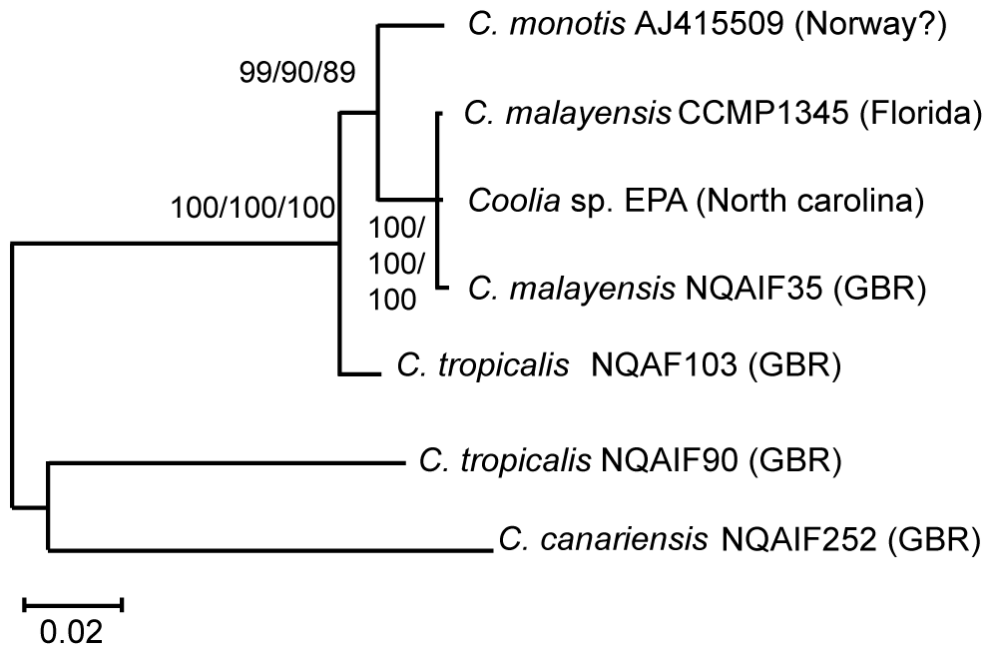
Phylogeny of the genus *Coolia* based on near-complete 18S rRNA gene sequences. Branch labels indicate Bayesian clade credibility values, and MP and ML bootstrap support, respectively. The tree was midpoint rooted. Scale bar represent number of changes.

The final alignment used for phylogenetic reconstruction and based on 22 partial 28SrRNA sequences included 398 sites, of which 174 were parsimony-informative. The final alignment of the seven near-complete 18S rRNA sequences included 1550 sites, of which 91 were parsimony-informative. For both gene regions, Bayesian inference (BI), maximum parsimony (MP) and maximum likelihood (ML) analyses generated trees with identical topology. The phylogeny based on the 28S rRNA alignment shows six distinct and well supported clades of *Coolia*, four of which include isolates from the central GBR (Figure 1). Five of the six clades represent named species, *C. monotis* (Clade I), *C. malayensis* (Clade II), *C. canariensis* (Clade IV and Clade V) and *C. tropicalis* (Clade VI). Clade III represents the culture NQAIF103. Strains of *C. canariensis* (VGO775, 786 and 787) were found to belong to two distinct, well supported clades, suggesting the existence of a cryptic species. As the holotype of *C. canariensis* was based on the strain VGO787 [14], clade V is representative of *C. canariensis* while clade IV (to which the strain isolated from the GBR belongs) could represent a closely related cryptic taxon. The phylogenetic tree shows three closely related taxa, *C. monotis*, *C. malayensis* and the strain NQAIF103, with pairwise inter-clade p-distances of less than 0.15 (Figure 1 and Table 1). Members of Clade V (*C. canariensis*) and Clade IV are also very closely related with p-distances of 0.11 based on 28S gene sequences (Table 1). Other species or groups of species are connected by long branches, with inter-specific p-distances of between 0.27 and 0.30 (Table 2). The phylogeny reconstructed using the 18S rRNA gene sequences supports the 28S results, but p-distances between species are smaller, because the rate of evolution of this gene is slower (Figure 2 and Table 1). The phylogeny based on the 18S sequences gives a less comprehensive picture of this genus, as only a few sequences are available for phylogenetic reconstruction. In both 18S and 28S gene analyses, there is a clear gap between values of intra and inter-clade p-distances, indicating that both loci are suitable for determining species-level divergence within this taxon (Tables 1 and 3). This is consistent with previous studies which highlighted the power of rRNA sequences to identify species boundaries in dinoflagellates [33,34].

**Table 1:** Uncorrected p-distances (± S.E.) between clades of *Coolia* spp.

	**Clade I**	**Clade II**	**Clade III**	**Clade IV**	**Clade V**	**Clade VI**
**Clade I**		0.074±0.013	0.117±0.017	0.291±0.023	0.288±0.023	0.286±0.023
**Clade II**	0.024±0.004		0.120±0.017	0.285±0.023	0.288±0.023	0.286±0.022
**Clade III**	0.025±0.004	0.025±0.004		0.259±0.022	0.275±0.022	0.292±0.023
**Clade IV**	n/c	n/c	n/c		0.11±0.016	0.303±0.023
**Clade V**	0.107±0.008	0.122±0.009	0.114±0.008	n/c		0.301±0.023
**Clade VI**	0.114.008	0.112±0.008	0.107±0.008	n/c	0.114±0.008	

Values above the diagonal refer to the 28S rRNA gene and those below to the 18S rRNA gene.

**Table 2:** Primers used for amplification and sequencing of 18S and 28S rRNA genes.

**Primer**	**Primer sequence**	**Target region**	**Direction**	**Ta**
D1R^a^, ^f^	ACCCGCTGAATTTAAGCATA	28S rRNA	Forward	48 °C
1483R^b, h^	CTACTACCACCAAGATCTGC	28S rRNA	Reverse	48 °C
1256R^b, g^	GGTGAGTTGTTACACACTCC	28S rRNA	Reverse	50 °C
D2CF^b^, ^g^	CTTGAAACACGGACCAAGG	28S rRNA	Forward	50 °C
D3B^c^, ^g^	TCGGAGGGAACCAGCTACTA	28S rRNA	Reverse	50 °C
18ScomfF1^d, f^	GCTTGTCTCAAAGATTAAGCCATGC	18S rRNA	Forward	55 °C
18ScomR1^d, f^	CACCTACGGAAACCTTGTTACGAC	18S rRNA	Reverse	55 °C
18S1155R^b, g^	GTTGAGTCAAATTAAGCCGCAG	18S rRNA	Reverse	55 °C
18S970F^b, g^	CGAAGACGATYAGATACCGTC	18S rRNA	Forward	55 °C
Lp1F1^c^, ^f^	GTCCCTGCCCTTTGTACAC	ITS	Forward	52 °C
25F1R^e, f^	ATATGCTTAAATTCAGCGG	ITS	Reverse	52 °C

^a^ [54] , ^b^ this study, ^c^ [55], ^d^ [56], ^e^ [57] , ^f^ used for amplification and sequencing, ^g^ sequencing primer, ^h^ amplification primer

**Table 3:** Within clade uncorrected p-distances (± S.E.) .

	**Clade I**	**Clade II**	**Clade III**	**Clade IV**	**Clade V**	**Clade VI**
**18S**	n/c	0.000±0.000	n/c	0.000±0.000	n/c	n/c
**28S**	0.002±0.001	0.000±0.000	n/c	0.000±0.000	0.000±0.000	0.025±007

**Figure 3:**
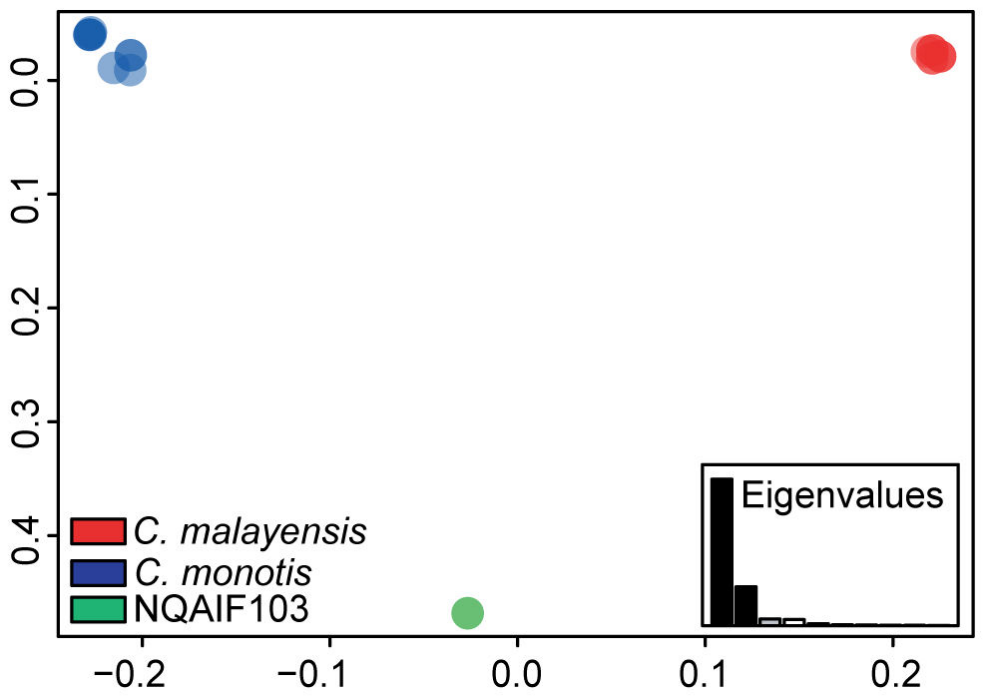
PCoA of the ITS distance matrix. Symbols are partially transparent to enable visualisation of higher density of points via colour saturation.

The final alignment (19 sequences) of the ITS region included 368 positions, of which 140 were variable and 78 were parsimony-informative. P-distances between species were high, varying from 0.206±0.02 (*C. monotis* vs. *C malayensis*) to 0.305±0.025 (NQAIF103 vs. *C. malayensis*), while intra-specific distance was highest in *C. monotis* (0.022±0.005) (Table 4, Figure 3). Inter-cluster p-distances were one order of magnitude higher than the cut-off value (P>0.04) previously proposed by Litaker [35] as diagnostic of species-level divergence. A graphical representation of the distance matrix by Principal Coordinates Analysis, (PCoA) (Figure 3) clearly shows the extent of the between-group divergence, when compared to within-species distance.

**Table 4:** ITS uncorrected p-distance (± S.E.) between NQAIF103 and the closely related *C. monotis* and *C. malayensis*.

	***C. monotis* N=9**	***C. malayensis* N=9**	**NQAIF103**
***C. monotis***	*0.022±0.005* ^*a*^		
***C. malayensis***	0.206±0.02	*0.004±0.002 * ^*a*^	
**NQAIF103**	0.294±0.023	0.305±0.025	*n/c ^a^*

a Indicate within-species distance.

### Morphological description of *Coolia sp*. NQAIF103

**Figure 4:**
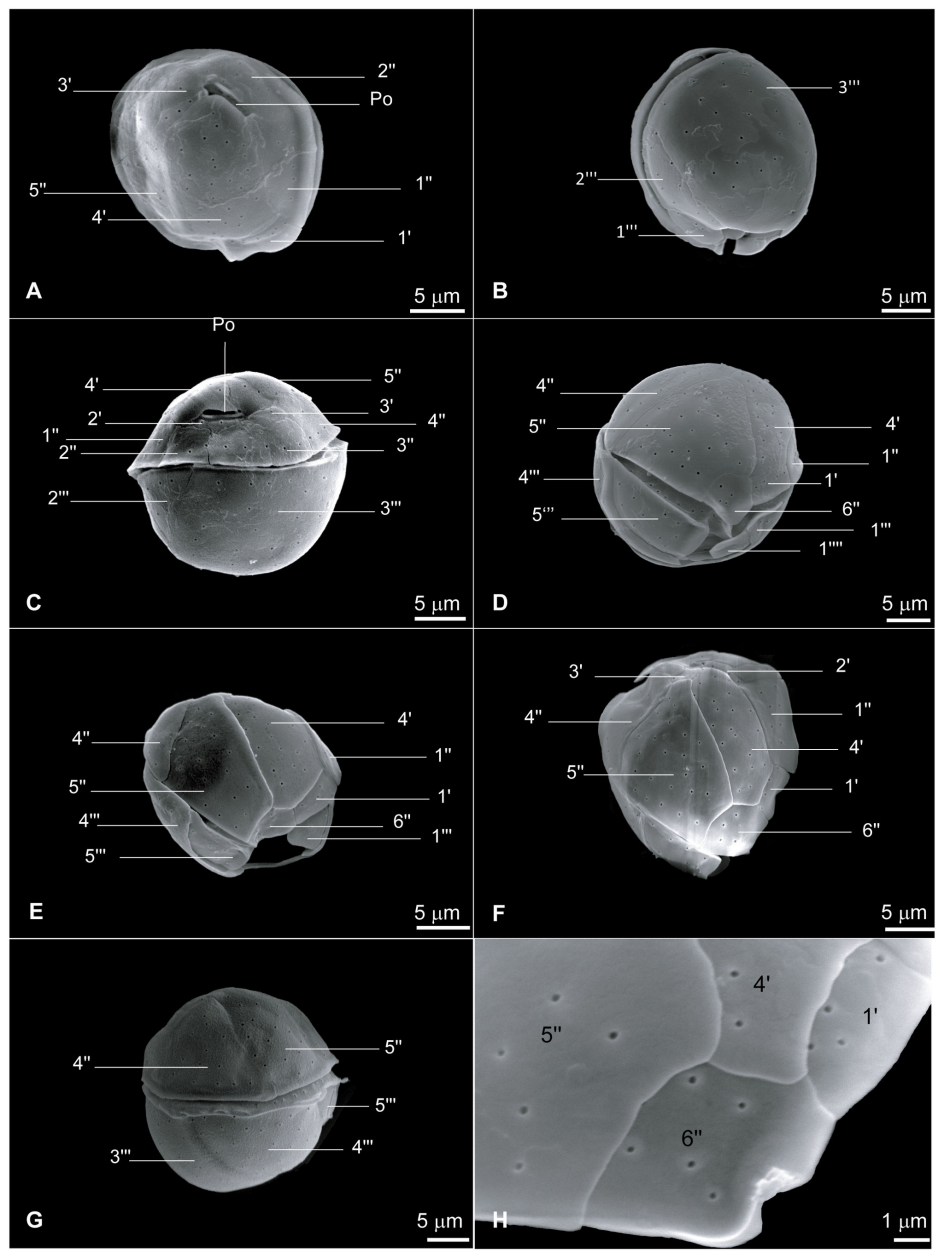
Scanning electron micrographs showing plate arrangements of NQAIF103 , following Besada's plate notations [36]. A: apical view, B: oblique left-antapical view, C: dorsal view, D: ventral view, E: ventral view of the epitheca, F: collapsed epitheca, G: left lateral view, H: detail of the 6'' plate.

**Figure 5:**
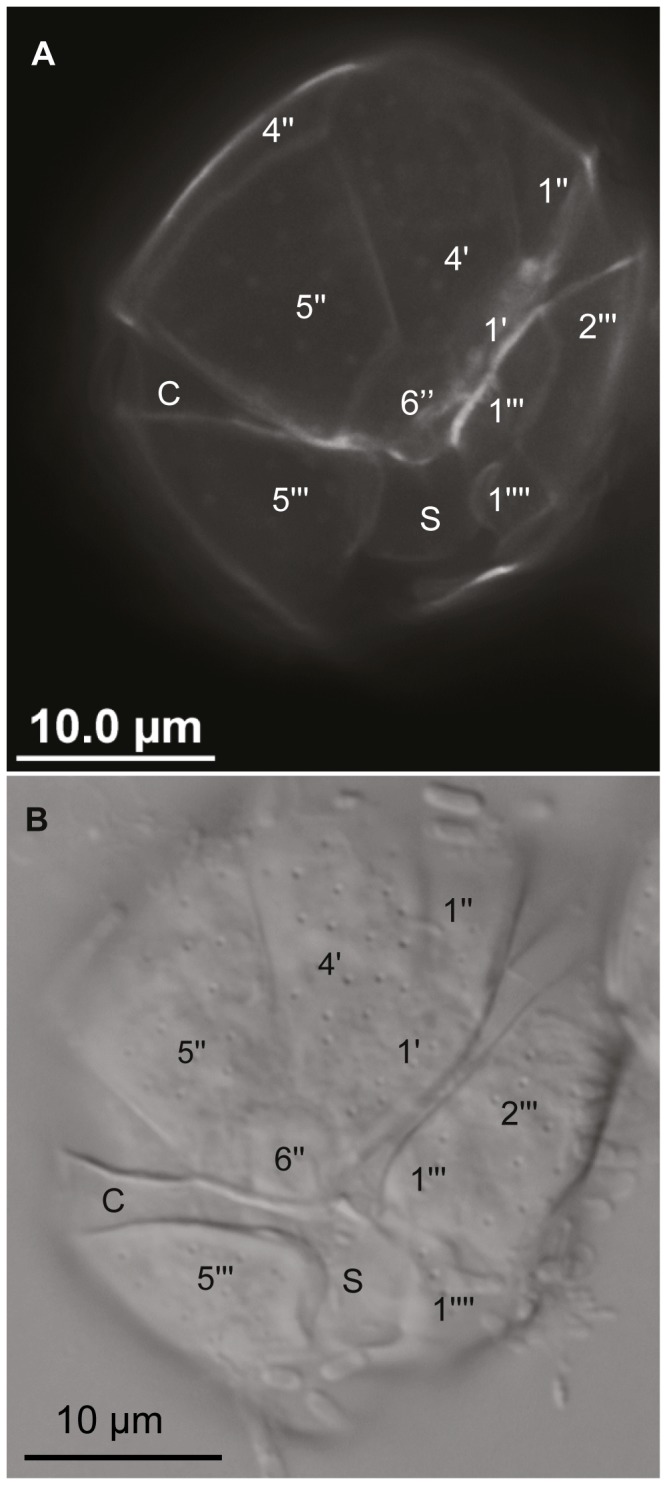
Calcofluor white epifluorescence micrograph (A) and differential interference light micrograph (B) of NQAIF103 showing ventral plate configuration.

**Figure 6:**
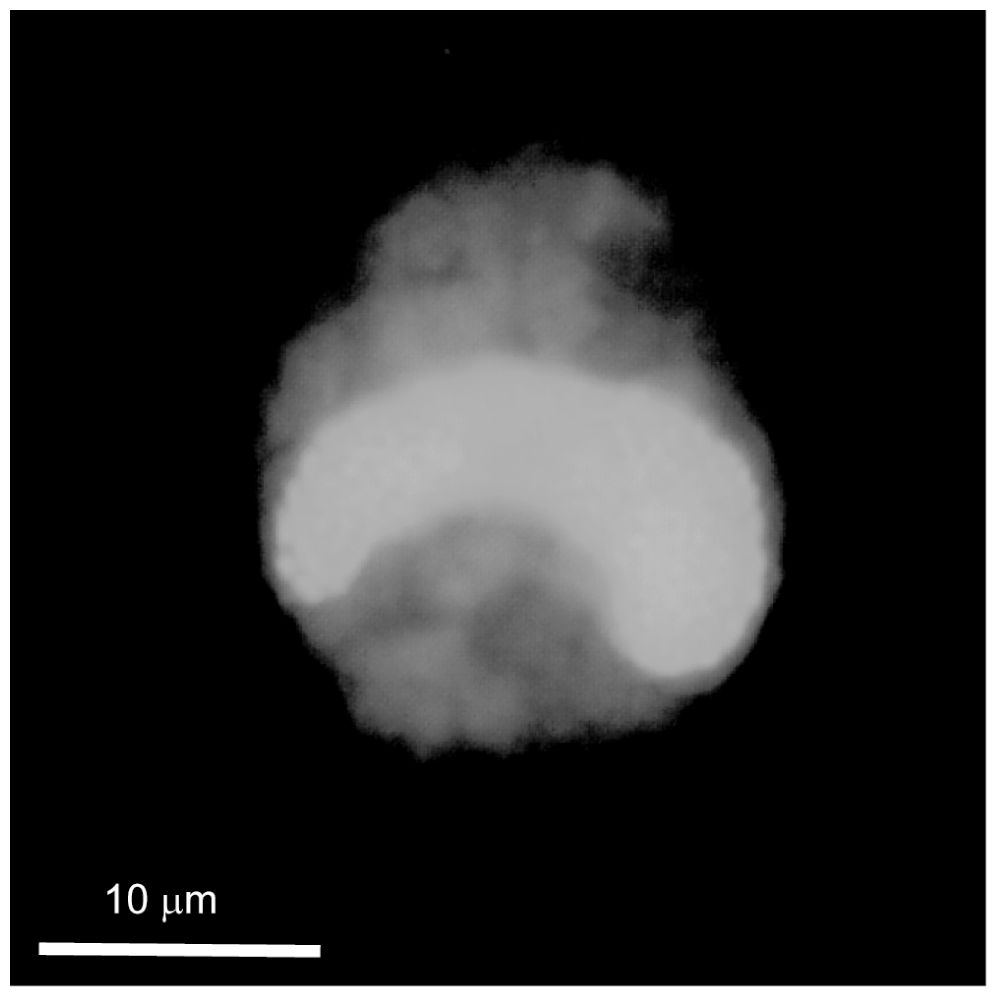
SYBR Green epifluorescence micrographs showing the U-shaped nucleus of NQAIF103.

While phylogenetically very distinct, the strain NQAIF103 was found to be morphologically nearly indistinguishable, based on published descriptions, from *C. malayensis* and *C. monotis*. Cells are more or less spherical, with a smooth surface covered in irregularly scattered pores (at an average distance of 2±1 µm, n = 10). NQAIF103 has the smallest cell size of any strain of *Coolia* thus far isolated: the antapical / apical axis is 19 to 23 µm long (average length = 21.8±1.5 µm, n = 10) and cells are 17 to 22 µm wide (average width = 18.9±2.3 µm, n = 10) and 18 to 23 µm thick (average: 20.2±1.9, n = 14). The plate tabulation (following Besada's plate notation [36]) follows the same formula as for other *Coolia* species: Po, 4', 6'', 6c?, ?S. 5''', 2''''. The apical pore is straight and approximately 2.8 to 4.7 µm long (Figure 4A, average: 4±0.6 µm, n = 7), therefore shorter than in any strain of *Coolia* isolated in the past [32]. Plate 4' is elongated and pentagonal in apical view (Figures 4A and 4F) but appears elongated and narrow in ventral view (Figures 4B, 5A and 5B). Plate 4' borders the apical pore complex (Po), plates 3', 2', 5'', 6'', 1', 1'' (Fig 4F). The 2' plate is inconspicuous, elongated and narrow (Figure 4A). Plate 5'' is the largest plate of the epitheca (Figures 4D, 4E, 4F). Plate 6'' has a width to length ratio between 0.8:1 and 1.2:1 (Figures 4D, 4H, 5A and 5B n = 6). The nucleus is U-shaped, equatorial, dorsally located with the arms pointing ventrally (Figure 6). 

### Morphological description of *Coolia sp*. strain NQAIF90

**Figure 7:**
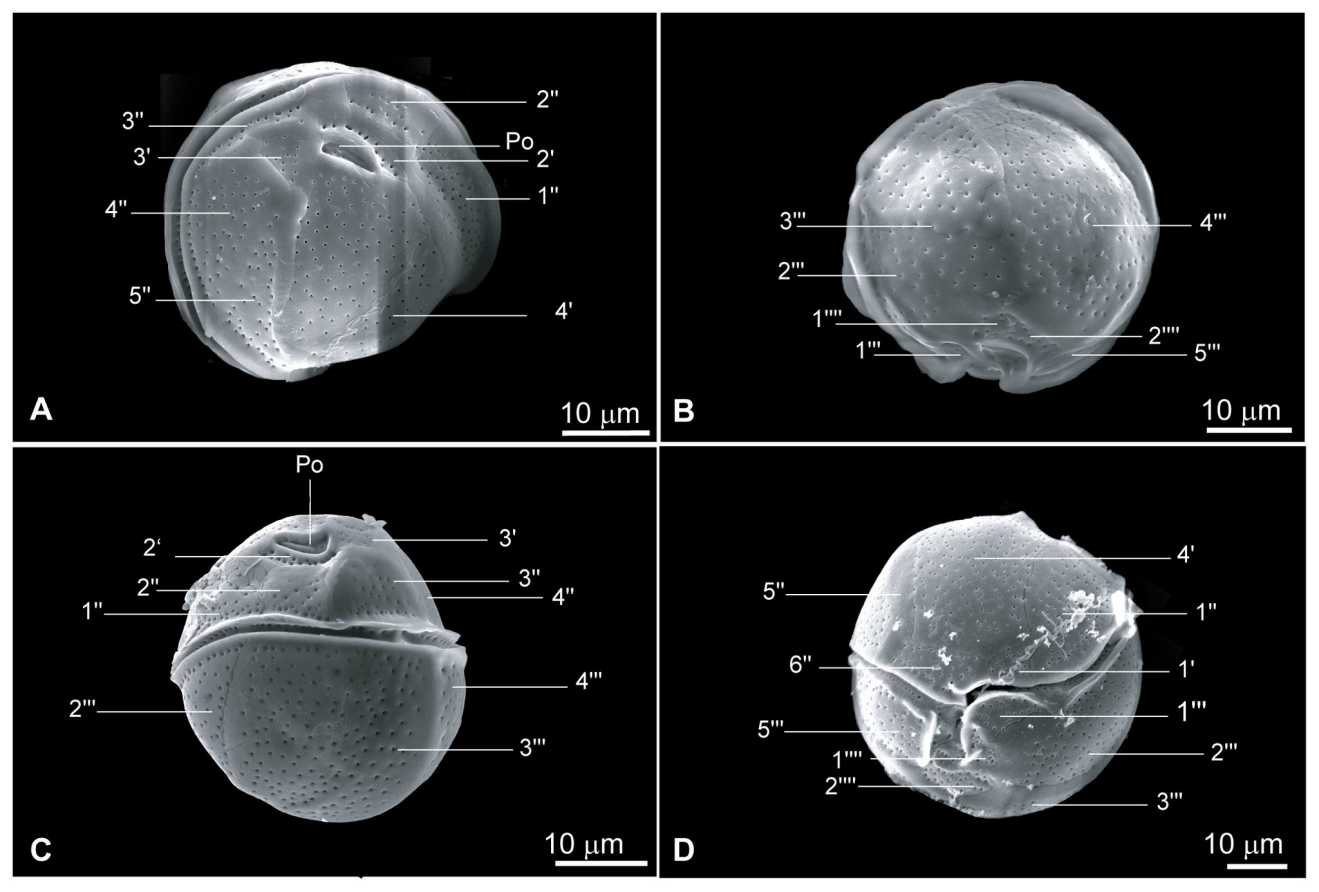
Scanning electron micrographs showing plate arrangements of *Coolia tropicalis* (NQAIF90), following Besada's plate notations [36]. A: apical view, B: antapical view, C: dorsal view, D: ventral view, .

The morphology of cells in the culture NQAIF90 matches very closely a recent re-description of *C. tropicalis* [32] and we regard NQAIF90 as belonging to that species. Cells are almost spherical when observed in antapical / apical and dorso / ventral views. Cells are 33 to 42 µm long (average: 39.04±2.94 µm, n = 12), 33 to 40 µm wide (average: 38.32±3.34 µm, n = 11) and 35 to 42 µm thick (thickness measured as the length of the dorso-ventral axis, average: 37.9±3.2µm, n = 11). The cell surface is smooth and covered with numerous regularly spaced circular pores at an average distance of 1.6±0.6 µm (n=10). The plate tabulation (following Besada's plate notation [36]) follows the formula: Po, 4', 6'', 6C?, ?S, 5''', 2''''. The pentagonal 4' plate is the largest plate of the epitheca (Figures 7A, 7D). The apical pore (Po) appears straight in apical view, 6.5 to 8 µm long (Figure 7A, average: 7±0.62 µm, n = 7). The 6'' plate is wide and short, with a width to length ratio between 2:1 and 4:1 (Figure 7D, n = 6). The 1''' plate is very small (Fig. 7D) and difficult to identify in antapical view (Fig. 7B). The 2''' plate is also small and variable in size (Fig. 7B). The 5''', 1''' and 1'''' plates bear wing-like extensions on the edges bordering the sulcus (Figure 7D). The cingulum also bears lists (Figure 7D). 

## Discussion

The central GBR harbours a high diversity of *Coolia* species: of the five species that have been described to date, three (*C. tropicalis*, *C. malayensis* and *C. canariensis*) occur in these waters. The strain of *C. canariensis* isolated from the GBR may in fact be a cryptic species, closely related to but nevertheless phylogenetically distinct from the strain on which the holotype of *C. canariensis* was based. Furthermore, a cryptic species morphologically very similar to both *C. malayensis* and *C. monotis* is reported here for the first time.

### Comparison of NQAIF103 with other *Coolia* spp.

Several features permit morphological discrimination of this strain from other species of *Coolia*. Firstly, with an average length of just over 20 µm and an average width of 19 µm, this Australian strain has smaller cells than any of the species that have been described thus far. Morphologically it is very similar to *C. monotis* and *C. malayensis*: plate 5'' is the largest plate of epitheca, plate 4' is long and narrow, cells lack ornamentation and the width to length ratio of plate 6'' is approximately 1. NQAIF103 does not seem to have the fine within-pore perforations that have been observed in *C. malayensis* [11,17], and pores appear to be more sparse than in previous descriptions of *C. monotis* [11,37]. Despite being morphologically similar to both *C. monotis* and *C. malayensis*, the extent of genetic divergence suggests that there is a long-lasting reproductive barrier between this clade and other *Coolia* species. Litaker [35] investigated species level divergence of the ITS in dinoflagellates, and found that uncorrected p-distances of ≥ 0.04 can be used to delineate most dinoflagellate species. Divergence of ITS sequences has been used for delineating new dinoflagellate species when morphological differences are small, such as the case of *C. malayensis* [17] and *Gambierdiscus ruetzleri* [18]. Yao et al. [38] reported similar (but slightly higher) divergence rates across the plant and animal kingdom for ITS2 sequences, and proposed that ITS2 should be used as a universal barcode for plants and animals. In our study we used the entire ITS region (including the more conserved ITS1, the very highly conserved 5.8S gene and the ITS2), and determined that the strain NQAIF103 is separated from its closest relative (*C. monotis*) by a p-distance of nearly 0.3. This is one order of magnitude higher than the highest within-species ITS2 p-distance reported by Litaker, and one order of magnitude higher than within-species ITS2 p-distances reported for any animal or plant species by Yao et al. [38]. Furthermore, *C. monotis* seems to be geographically restricted to the temperate Mediterranean Sea and the East Atlantic (see next sections), its range being nearly at the antipodes of the tropical location from which NQAIF103 was isolated. We conclude that the combination of morphological differences, genetic analysis of three rRNA markers and geographical isolation suggest that this strain likely represent a new cryptic species. 

### Morphology and genetics

In addition to NQAIF103, a further cryptic species might be discernible using this data: the 28S divergence between clades IV and V is high (p distance = 0.11±0.016), greater, for example, than between the two closely related species *C. malayensis* and *C. monotis*. It is possible that these two clades represent in fact two distinct species, but the lack of morphological information for the strain VGO775 [14] hinders the assessment of this hypothesis. Strain VGO787 is the type strain for *C. canariensis* and thus this name must remain with members of Clade V should future work demonstrate that Clade IV represents a distinct species. 

Two morphological traits that seem consistent with molecular phylogenetic reconstructions are the size and shape of the 4' and 6'' plates. Plate 6'' width to length ratio in particular has been proposed as a stable morphological feature, which allows differentiation between *Coolia* species [14]. *Coolia monotis* has a width/length ratio of around 1 for this plate, for *C. areolata* and *C. canariensis* the value is around 2 and around 4 for *C. tropicalis* [11]. In the closely related *C. monotis*, *C. malayensis*, and NQAIF103, the 4' plate is narrow and elongated and is not the largest plate of the epitheca, while the 6'' plate is short, with a low width to length ratio [17,37,39]. In *C. tropicalis* (NQAIF90), *C. canariensis* and *C. areolata* the 4' plate is beret-shaped and occupies most of the epitheca, while the 6'' plate is larger and with a higher width to length ratio than in other species [14,31]. While no sequence data are available for *C. areolata*, it seems likely that this species is more closely related to *C. tropicalis* and *C. canariensis* based on morphology. It should also be noted that the only two species exhibiting areolation are *C. areolata* and *C. canariensis.*

### Taxonomic confusion and the diversity of *Coolia*

This study supports the identification of *C. monotis* as the original European clade. Notably, every *Coolia* sampled outside of the East-Atlantic and Mediterranean Sea, and initially thought to represent *C. monotis*, was subsequently identified as a new species [14,17,30,31], supporting the hypothesis that *C. monotis* is not a cosmopolitan species, but is geographically restricted. The considerable morphological similarities between *C*. *monotis* and the widespread *C. malayensis* as well as NQAIF103, described in this study, may account for previous misidentifications. Adding to the confusion, the first thorough morphological description of “*C. monotis*” based on SEM observations used a sample from Belize [37]. Some morphological differences between this Belizean strain and the European strain , including the differences in the distance between pores, are noticeable [37]. Furthermore the width to length ratio of plate 6'' of the specimen from Belize is clearly not around one as in other strains of *C. monotis* (see Figure 6 in [37]). This Belizean strain was not deposited in any culture collection or museum and no phylogenetic analysis was performed, hindering more thorough morphological examinations and genetic analysis. 

Cooliatoxin was extracted in 1998 from an Australian strain originally identified as *C*. *monotis* [9], leading to the assumption that *C*. *monotis* is a toxic species. However, the Australian strain was later identified as *C. tropicalis* [32]. There is no published study that actually shows toxicity in the European *C. monotis*. In a recent study, *C. canariensis* and *C. monotis* were cultured from macroalgal substrates obtained from the south-eastern Bay of Biscay and toxicity tests showed that these strains were non-toxic to the crustacean *Artemia franciscana* [11]. Similarly, other toxicity studies used strains from New Zealand and the Cook Islands (CAWD39, CAWD151) [26,27]. Both of these were first identified as *C. monotis*, as at the time the studies were conducted, the species *C. malayensis* had not been described. Based on 28S rRNA sequences, the toxic strains CAWD151 and CAWD39 grouped with the “*malayensis*” type [17,26]. It thus appears that *C. monotis* might be harmless, while both *C. tropicalis* and *C. malayensis* are likely harmful. Given that within-strain toxicity levels can vary, but the reason for such variability is presently unknown [10,11], it is important to screen several strains of each species, raised under the same environmental conditions, for toxicity. At present, toxicity status of the strain NQAIF103 described in this study is unknown. *Artemia*-based toxicity assays are under way for cultured and freshly isolated strain to clarify this issue. 

Four cultures of *Coolia* spp. established from samples obtained from the central GBR represented four distinct phylogenetic clades (Figure 1). Strains of *C. canariensis*, *C. malayensis* and *C. tropicalis* have now been sampled in the GBR as well as in other distant locations (*C. tropicalis* in Belize and Indonesia, *C. malayensis* in Malaysia and Florida and *C. canariensis* in the Canary Islands), suggesting these taxonomic groups have a very wide, transoceanic distribution, a notable contrast with the apparently geographically restricted *C. monotis*. The GBR is now the area from which most clades of *Coolia* have been isolated. This is consistent with biodiversity studies on other taxa (fishes, corals and other marine invertebrate), which suggest the Indo-Australian Archipelago as the most important marine biodiversity hotspot in the world [40,41].

## Materials and Methods

### Culture isolation and maintenance

Macroalgal samples were collected by Parks and Wildlife Queensland (no special permit required because it is the authority for issuing permits for this area) from Pallarenda at the mouth of Three Mile Creek (Latitude: 19° 12' 34'' S, Longitude: 146° 46' 36'' E; central GBR), following a brown discolouration of the water in August 2004, and delivered to the North Queensland Algal Identification/Culturing Facility (NQAIF) at James Cook University, Townsville, Queensland, Australia. Epiphytic microalgae were dislodged from the macroalgal substrata using filtered seawater. The suspended epiphytic microalgae were concentrated by sequential filtration through 60 μm and 20 μm nylon mesh filters, placed in a Petri dish and observed using an Olympus inverted microscope (CKX-41; Olympus Australia Ltd, Mt Waverley VIC 3149). Following the isolation procedures outlined below, these samples gave rise to cultures NQAIF90 and NQAIF103. Cultures of *Coolia* spp. (NQAIF35 and NQAIF252) were established from macroalgal-derived suspended materials. The macroalgae were obtained from Nelly Bay, Magnetic Island (19° 10’ S, 146° 50’ E) in July 2004 (Great Barrier Reef Marine Park Authority Permit G06/20234.1) and from Pioneer Bay, Orpheus Island (Great Barrier Reef Marine Park Authority Permit G10/33239.1; in front of the outdoor laboratory at the research station approximately 18° 37′ 06″ S 146° 29′ 37″ E) in March 2008, respectively. The *Coolia* culture NQAIF 60 was donated by Shauna Murray in July 2004, who established this culture from water samples collected in Fiji (no special permit requirements, as the location is neither privately owned, protected nor did sampling involve endangered or protected species). 

Individual cells, used to start cultures NQAIF35, 90, 103 and 252, were isolated by microcapillary by Stanley Hudson at 10x magnification on an inverted light microscope (Olympus CKX41). Cells were dispensed into autoclaved and filtered (0.45 μm, Durapore, Millipore) seawater, allowed to swim for ten minutes and were then recaptured. This procedure was repeated ten times to ensure that nano- and pico-plankton were no longer in the vicinity of the cell to be isolated. Cultures were established in autoclaved L1 medium [42] prepared in natural 0.45 µm filtered seawater and maintained at 24°C, a 12:12 h photoperiod and light intensity of 45 µmol photons m^-2^s^-1^ in a Contherm cross-flow phytoplankton growth chamber (Contherm Scientific Limited, Hutt City, NZ). Cultures continue to be sub-cultured in L1 medium every four weeks. 

### DNA extraction, amplification and sequencing

Cells from the five cultures of *Coolia* spp. (Table 5) were pelleted by centrifugation (2,300 RCF for 5 min, in an Eppendorf 5415D centrifuge, North Ryde, NSW 2113, Australia) washed 3 times in TE buffer (10 mM Tris-HCl,1 mM EDTA, pH 8) followed by the same centrifugation protocol before transfer to 1.5 mL Eppendorf tubes containing 500 µl of 10% Chelex^®^ 100 [43] and 5 µL of 20 mg mL^-1^ proteinase K. Tubes were incubated in a rotating oven for 2 hours at 55°C followed by incubation at 94°C for 20 min. Tubes were then centrifuged at 9,200 RCF (Eppendorf 5415D) for 5 min and the supernatant transferred to a new Eppendorf tube and stored at -20°C. PCR and sequencing of the near-complete 18S rRNA gene, a fragment of the 28S rRNA gene encompassing all or some of the D1-D6 regions and the full ITS region (the last for NQAIF103 only) used primers and annealing temperatures as listed in Table 2. PCR reactions were set up as follow: 1 μL of template DNA, 800 µM each dNTPs, 5 pmol each primer, 2.5-3.5 mM MgCl_2_, 1x Kapa2G PCR buffer B and 1 unit of Kapa2GFast (GeneWorks Pty Ltd, Hindmarsh, South Australia, Australia) in a total volume of 20 µL. Reactions underwent three min initial denaturation at 94°C, and 35 cycles of 30 s denaturation at 94°C, 15 s annealing (see Table 2 for annealing temperatures), and 60 s extension at 72°C, and a final extension step of 3 min at 72°C. PCR products were visualized on a 1.5% agarose gel, and cleaned by isopropanol precipitation. Sequencing reactions were performed at the Australian Genome Research Facility (University of Queensland) using Big Dye Terminator Cycle Kit v3.5 (Applied Biosystems, Mulgrave, Victoria Australia). Capillary separation was performed on an AB 3730xl platform.

**Table 5:** Strains of *Coolia* spp. used for phylogenetic reconstructions.

**Species**	**Strain**	**Source location**	**Locus**	**GenBank acc. n.**
*C. monotis*	UoA-Cmon1	Thermaikos Gulf, Greece	28S	EU477760
	UoA-Cmon3	Thermaikos Gulf, Greece	28S	EU477761
	VGO782	Saronikos Gulf, Greece	28S	AM902746
	VGO783	Saronikos Gulf, Greece	28S	AM902747
	VGO831	*Almeria*, Spain	28S	AM902744
	Cm7C	Catalan Sea, Spain	28S	AM902745
	RIKZ4	North Sea, Netherlands	28S	AM902749
	SZN268	Naples, Italy	28S	AM902748
	CBA1	Genoa, Italy	28S	AM902742
	?	Norway ?	18S	AJ415509
*C. malayensis*	CCMP1345	Florida, USA	18S/ 28S	EF492487/AM902743
	CmPL01	Malaysia	28S	AF244942
	NQAIF60^a^	Fiji	28S	HQ897275
	NQAIF35 ^a^	Magnetic Is., GBR	18S/28S	HQ897279, HQ897274
	CAWD39	New Zealand	28S	CMU92258
*C. canariensis*	VGO775	Tenerife, Canary Is.	28S	AM902739
	NQAIF252 ^a^	Orpheus Is., GBR	18S/28S	HQ897274, HQ897279
	VGO786	Tenerife, Canary Is.	28S	AM902737
	VGO787	Tenerife, Canary Is.	28S	AM902738
*Coolia* *sp.*	NQAIF103 ^a^	Pallarenda, GBR	18S/28S	HQ897277/HQ897281
*Coolia tropicalis*	NQAIF90 ^a^	Pallarenda, GBR	18S/28S	HQ897276/HQ897280
	CCMP1744	Twin Cay, Belize	28S	AM902741
	EPA	North Carolina, USA	18S	EF492488

a strains isolated in this study. All NQAIF strains were isolated by Stanley Hudson, with the exception of NQAIF60 (from Fiji) which was donated to the culture collection by Shauna Murray.

### Sequence alignment

Overlapping fragments of the 18S and 28S rRNA genes, as well as the full ITS region of NQAIF103, were assembled using the software Chromas Pro (Technelysium Pty Ltd., Tewantin, Queensland, Australia, http://www.technelysium.com.au/ChromasPro.html). Sequences obtained from cultures were aligned with available sequences of *Coolia* spp (Table 5) using ClustalW [44], and the alignment was subsequently refined by eye using BioEdit [45]. The D2 hypervariable region of the 28S rRNA gene was excluded from analysis, as the high number of indels in this region prevented unambiguous alignment. Regions downstream from the D2 region were also excluded from the analysis as these sequences were not available for any strains listed in Genbank (other than those sequenced in this study). The ITS region of NQAIF103 was aligned with the ITS sequences of the closely related *C. malayensis* and *C. monotis* available in GenBank (AJ491336-9, AF244950, AJ308524, AJ319578, AJ514919, AJ515260, AJ532583, AF244943-50)

### Sequence analysis

Between-species and within-species uncorrected p-distances were estimated using MEGA4 [46] for the 18S, 28S and ITS alignments, using 1000 bootstrapped data sets. The p-distance matrix obtained from the ITS alignment was analyzed with a Principal Coordinate Analysis (PCoA) performed in the R environment using the package Ade4 [47]. Two phylogenies of the genus *Coolia*, one based on the partial 28S rRNA gene and one based on the near-complete 18S rRNA gene, were inferred by Bayesian inference (BI), and by analysis of 1000 bootstrapped pseudo-replicated data sets by maximum parsimony (MP) and maximum likelihood (ML). BI analysis was performed in MrBayes 3.1.2 [48] using the GTR+I+Γ model. Initially the number of Monte Carlo Markov Chain simulations was set to 4,000,000, but the run was programmed to automatically stop when the standard deviation of split frequencies (as calculated for the last 75% of sampled trees) fell below 0.01. Trees were sampled every 100 generations. ML analysis was performed using the hill-climbing algorithm implemented by PHYML [49] on the online PHYML web server [50]. The analysis was performed using the GTR+I+Γ model, with 4 gamma parameters. Data were analysed by MP using the software package Phylip 3.6 [51,52]. Bootstrapped pseudo-replicated data sets for MP analysis were generated using SeqBoot [52]. Phylogenies based on 28S and 18S bootstrapped data sets were generated using DNApars, and consensus trees were produced using Consense [52]. The final tree topology and branch lengths presented are from BI analysis, and branch reliability is presented as Bayesian clade credibility values and MP and ML bootstrap support.

### Microscopy

The morphology of cultured cells was investigated by light-microscopy (LM), calcofluor white staining and epifluorescence microscopy (λ_ex_= 400 nm, λ_em_=500-520 nm) [53] and SEM. Light and epifluorescence microscopy were carried out using an Olympus BX51 microscope equipped with Nomarski differential interference contrast, epifluorescence optics, and a CCD-cooled digital camera DP70 (Olympus Australia Ltd , Mt Waverley VIC 3149). Cultures of *Coolia* spp. were identified to species level based on plate tabulation. Cells fixed in 4% formalin were stained with calcofluor white (Sigma-Aldrich; Castle Hill, NSW 1756, Australia) following the manufacturer’s instructions. For SEM, cells were fixed in 2% OsO_4_ or 2% gluteraldehyde (Sigma-Aldrich), followed by a gradual reduction in salinity by the addition of freshwater, and dehydrated in a graded series (10% dehydration steps) of ter-butanol or ethanol. A few drops of hexamethyldisilazane (Sigma-Aldrich) were then added, and the specimens mounted on stubs and air-dried overnight. Stubs were sputter-coated with gold and visualized on a JEOL JSM-5410LV scanning electron microscope (JEOL, Frenchs Forest NSW 2086, Australia) at the Advanced Analytical Centre at James Cook University. To determine the shape of the nucleus, cells fixed in 4% formalin were stained with 1X SYBR Green 1 (Invitrogen Australia Pty Limited, Mount Waverley, VIC 3149, Australia), mounted in Prolong Antifade (Invitrogen) and visualized by epifluorescence microscopy.
